# TRAF6 Mediates IL-1β/LPS-Induced Suppression of TGF-β Signaling through Its Interaction with the Type III TGF-β Receptor

**DOI:** 10.1371/journal.pone.0032705

**Published:** 2012-03-12

**Authors:** Seunghwan Lim, Eunjin Bae, Hae-Suk Kim, Tae-Aug Kim, Kyunghee Byun, Byungchul Kim, Suntaek Hong, Jong Pil Im, Chohee Yun, Bona Lee, Bonghee Lee, Seok Hee Park, John Letterio, Seong-Jin Kim

**Affiliations:** 1 Department of Pediatrics, Case Western Reserve University, Cleveland, Ohio, United States of America; 2 CHA Cancer Institute, CHA University, Gangnam-gu, Seoul, Korea; 3 Lee Gil Ya Cancer and Diabetes Institute, Gachon University of Medicine and Science, Songdo, Incheon, Korea; 4 Laboratory of Cancer and Stem Cell Biology, National Cancer Institute, Bethesda, Maryland, United States of America; 5 Department of Biochemistry, College of Natural Sciences, Kangwon National University, Chuncheon, Korea; 6 Department of Internal Medicine and Liver Research Institute, Seoul National University College of Medicine, 28 Yongon-dong, Chongno-gu, Seoul, Korea; 7 Department of Molecular Biology, Sungkyunkwan University, Suwon, Korea; Memorial Sloan Kettering Cancer Center, United States of America

## Abstract

Transforming growth factor-β1 (TGF-β1) is an important anti-inflammatory cytokine that modulates and resolves inflammatory responses. Recent studies have demonstrated that inflammation enhances neoplastic risk and potentiates tumor progression. In the evolution of cancer, pro-inflammatory cytokines such as IL-1β must overcome the anti-inflammatory effects of TGF-β to boost pro-inflammatory responses in epithelial cells. Here we show that IL-1β or Lipopolysaccharide (LPS) suppresses TGF-β-induced anti-inflammatory signaling in a NF-κB-independent manner. TRAF6, a key molecule in IL-1β signaling, mediates this suppressive effect through interaction with the type III TGF-β receptor (TβRIII), which is TGF-β-dependent and requires type I TGF-β receptor (TβRI) kinase activity. TβRI phosphorylates TβRIII at residue S829, which promotes the TRAF6/TβRIII interaction and consequent sequestration of TβRIII from the TβRII/TβRI complex. Our data indicate that IL-1β enhances the pro-inflammatory response by suppressing TGF-βsignaling through TRAF6-mediated sequestration of TβRIII, which may be an important contributor to the early stages of tumor progression.

## Introduction

Transforming growth factor-(TGF-β) is an important cytokine that plays fundamental roles in regulating cell proliferation, apoptosis, differentiation, and migration [1]. In the canonical signaling pathway, the TGF-β ligand directly binds to TβRII or TβRIII which, in turn, phosphorylates and activates TβRI. The activated TβRI recruits and phosphorylates Smad2 and Smad3 (R-Smads). These R-Smads form a complex with Smad4 which translocates into the nucleus to regulate TGF-β target genes [2]. TβRIII was originally described as an accessory receptor because it has no known enzymatic activity in its cytoplasmic domain and seemed to enhance TGF-β signaling by simply presenting TGF-β ligands to TβRII [3]. However, growing evidence indicates that TβRIII has a more complex role in the regulation of TGF-β signaling especially in cancer [4], [5].

Aberrant TGF-β signaling, often caused by functional loss of key signaling components, is found in a broad range of cancers. Perturbation of TGF-β signaling, which most commonly results in a loss of its growth inhibitory function, provides a favorable condition for early-stage tumors to progress into malignant cancer. Therefore, it is important to identify the regulatory mechanisms of TGF-β signaling in the early stages of cancer development.

Persistent inflammation is closely linked to cancer progression through its stimulation of cell proliferation, survival, invasion, and metastasis [6], [7]. In normal tissue, inflammation induced by pro-inflammatory cytokines (e.g. IL-1β and TNF-α) is tightly regulated by anti-inflammatory cytokines (e.g. IL-10, IL-13, and TGF-β), which resolve the inflammatory response [8]. Therefore, it is essential to understand the mechanisms by which these two classes of cytokines function in a coordinate manner to maintain cellular homeostasis.

Pro-inflammatory mediators such as IL-1β and LPS activate NF-κB and p38/JNK pathways through the TRAF6/TAK1 axis. It is well established that IL-1β and TGF-β have antagonistically regulating mechanism towards one another. For example, TGF-β inhibits IL-1β signaling by disrupting the Pellino1/IRAK1 complex by Smad6 [9], whereas TGF-β may block TNF-α signaling through the interruption of TRAF2/TAB2 and TAB3 association by Smad7 [10]. Conversely, pro-inflammatory cytokines are theorized to attenuate anti-inflammatory signals in order to enhance inflammatory responses. Indeed, it has been suggested that IL-1β and TNF-α exert suppressive effects on TGF-β-mediated Smad2/3 phosphorylation and Smad3/4-DNA binding [11]. Also, IL-1β has been shown to inhibit Smad3-mediated TGF-β target gene activation through its downstream effecter TAK1 [12]. NF-κB/RelA-mediated Smad7 induction has also been suggested as a possible means by which cells suppress TGF-β/Smad signaling [13]. Nonetheless, the underlying mechanism by which IL-1β attenuates anti-proliferative or apoptotic TGF-βsignaling is not fully understood.

In this study, we demonstrate that IL-1βor LPS is able to suppress TGF-β/Smad signaling through regulation of the TGF-β receptor complex. We show that TRAF6 binds to TβRIII in a TβRI kinase-dependent manner, and thereby suppresses TGF-β-Smad2/3 signaling.

## Results and Discussion

### IL-1β and LPS suppress TGF-β1-Smad2/3 pathways through TRAF6

**Figure 1:**
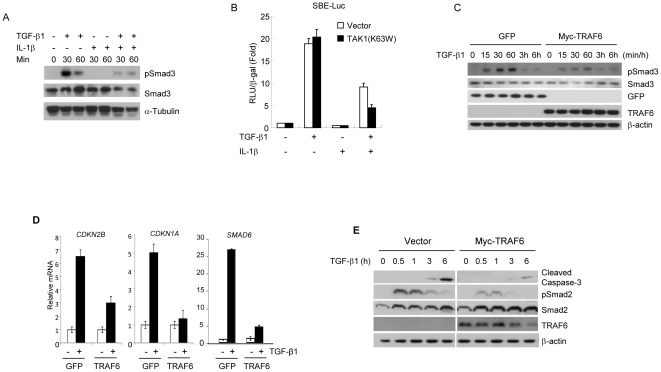
TRAF6 mediates IL-1β or LPS-induced suppression of TGF-β1/Smad pathway. (**A**) HEK293 cells were treated with TGF-β1 (0.4 ng/ml) and/or IL-1β (2 ng/ml) as indicated. TGF-β-mediated Smad3 phosphorylation was demonstrated by anti-pSmad3 and total Smad3 antibodies. As a loading control, α-tubulin was used. (**B**) SBE-Luc assay was performed in HepG2 cells. These luciferase assays were normalized by the activities of co-transfected β-galactosidase. (**C**) TRAF6 or GFP was over-expressed in HEK293 cells by use of a lentiviral system. Cells were harvested after TGF-β1 addition for up to 6 hours followed by westernblotting to compare phospho-Smad2/3 levels. (**D**) TGF-β1 target genes, *CDKN2B*, *CDKN1A*, and *SMAD6*, were detected by quantitative RT-PCR using total RNA from vector-(GFP) or TRAF6-expressing HaCaT cells treated as indicated. Human *GAPDH* was used as a loading control. (**E**) FaO cells were infected with either control vector or Myc-TRAF6 on previous day and then treated with TGF-β alone or together with LPS up to 8 hours. Both floating and adherent cells were harvested to compare the induction of cleaved caspase-3. TGF-β-induced signal transduction was displayed by showing pSmad2 level. The results are representative of three independent experiments.

In order to examine whether pro-inflammatory effectors (IL-1β or LPS) are able to suppress Smad2/3-mediated TGF-β1 signaling, we treated various cells with TGF-β1 in the presence or absence of IL-1β or LPS. Co-treatment of cells with TGF-β1 and IL-1β resulted in decreased phospho-Smad2/3 in HEK293 (Figure 1A) as well as in other cell lines such as HaCaT, 67NR, and FaO cells (Figure S1A, S1F, and S2A). We first examined whether TAK1, a key enzyme in pro-inflammatory signaling, is involved in IL-1β/LPS-mediated suppression of TGF-β signaling by using mutant TAK1 (K63W) which blocks IL-1β-mediated NF-κB activation (Figure S1B). TAK1 (K63W) failed to abrogate the IL-1β effect on TGF-β1/Smad signaling in SBE-luciferase reporter gene assay (SBE-Luc) containing promoter with Smad-binding sequences(Figure 1B). It has been reported that NF-κB-mediated Smad7 induction is one of the inhibitory mechanisms for TGF-β signaling [13]. However, one hour treatment with IL-1β did not change the messenger RNA levels of *SMAD7* in vector control, TRAF6, or TAK1 (K63W)-expressing cells (Figure S1C). TRAF6 is an upstream molecule of TAK1 in IL-1β and LPS pro-inflammatory pathway, and we therefore investigated TRAF6 as a candidate molecule mediating the IL-1β-induced inhibitory effect. It is important to note that over-expressed TRAF6 may form self-associated and auto-ubiquitinated TRAF6, which is constitutively active, mimicking IL-1β or LPS stimulation [14], [15]. TRAF6 expression in HEK293 cells suppressed TGF-β1-induced Smad3 phosphorylation as compared tocontrol cells overexpressing GFP (Figure 1C). To find out if Smad7 is required for TRAF6-mediated suppression of Smad3 transcriptional activity, we performed SBE-luc reporter gene assay in HepG2 cells with knock-down of Smad7. The elevated SBE reporter activityin response to TGF-β represented effective knock-down of Smad7. Under this condition, TRAF6 was able to suppress SBE reporter activity by showing 65% and 70% reduction in control cells (pLKO) treated with TGF-β and Smad7 knock-down cells (shSmad7) treated with TGF-β, respectively. (Figure S1D). This finding implies that TRAF6 may similarly suppress TGF-β/Smad3 signaling even in the cells with reduced Smad7 expression. Near to complete knock-down of Traf6 was achieved in 67NR mouse mammary epithelial cells via infection with lentivirus encoding Traf6 specific shRNA. In sh-Traf6 cells, LPS-mediated suppression of Smad3 activity disappeared as compared to the control sh-Luc counterpart (Figure S1E), indicating that TRAF6 might be a factor responsible for mediating the suppressive effect of LPS on TGF-β1-mediated Smad3 phosphoryation. HaCaT cells, due to the sensitivity to TGF-β-induced cell growth inhibition, were used to examine whether TRAF6 overexpressing cells give rise to reduction of TGF-β1 target genes. The results of both quantitative PCR and RT-PCR indicated that TRAF6 overexpressing cells were less sensitive to TGF-β-mediated induction of the cell cycle inhibitors such as *CDKN2B* and *CDKN1A* and *SMAD6* (Figure 1D and Figure S1F). These results further support the importance of TRAF6 as a critical mediator of IL-1β-induced suppression on TGF-β signaling. The induction of apoptosis in rat hepatoma FaO cells by TGF-β is known to be mediated by active Smad2/3 [16]. Thus, the biological significance was addressed by examining the suppressive role of pro-inflammatory signaling on TGF-β-induced apoptosis in FaO cells. The TRAF6 over-expressing FaO cells were less sensitive to TGF-β-induced apoptosis as evidenced by decreased cleaved-caspase3 and Smad2 phosphorylation (Figure 1E). Induction of cleaved-caspase 3 and Smad3 phosphorylation by TGF-β1 were decreased in LPS co-treated cells (Figure S2A). To further investigate whether the LPS-induced inhibition of TGF-β-induced apoptosis is dependent on NF-κB-mediated signaling, FaO cells were infected by adenovirus containing either LacZ or IκBαSR. IκBαSR is asuper-repressor mutant of IκBαwhich cannot be phosphorylated or degraded by NF-κB-activating stimuli, and eventually prevents NF-κB transcriptional activation [17]. Induction of cleaved-caspase 3 by TGF-β1 was not affected in the presence of IκBαSR, indicating that NF-κB-dependent survival factors are less likely to play a role in this anti-apoptotic event (Figure S2B). Moreover, apoptotic induction by TGF-β was significantly suppressed by LPS co-treatment as demonstrated by TUNEL and DNA fragmentation assay (Figure S2C and S2D). These results indicated that LPS may facilitate cell survival by suppressing Smad2/3 signaling pathways, possibly through TRAF6.

### Traf6 −/− primary cells showed elevated sensitivity to TGF-β1

**Figure 2:**
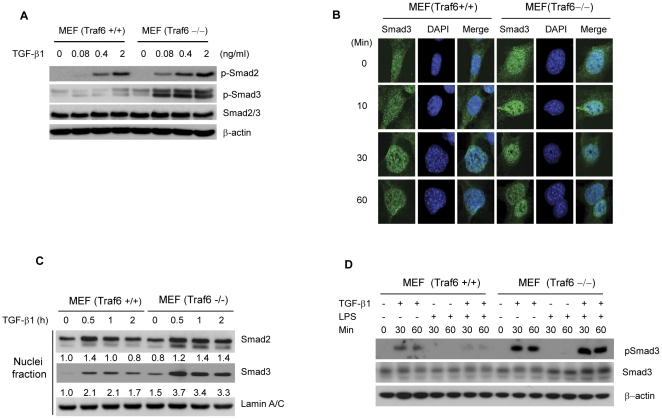
*Traf6*-deficient primary cells show a higher sensitivity to TGF-β1.

The physiological function of Traf6 in TGF-β1-Smad2/3 signaling was further investigated in Traf6+/+ and Traf6−/− mouse embryonic fibroblasts (MEFs). Messenger RNA and protein expression of TGF-β receptors in Traf6+/+ and Traf6−/− MEFs appeared to be similar in both MEF cells (Figure S3A). When we examined the sensitivity to TGF-β using the SBE-luciferase assay, Traf6−/− MEFs showed stronger responsiveness to TGF-β than the Traf6+/+ MEFs (Figure S3B). The sensitivity to TGF-β was further examined by observing the dose-dependent responsiveness. Consistent with the reporter assay, Smad2 and Smad3 phosphorylation was detected at the lowest concentration of TGF-β1 (0.08 ng/ml) in Traf6−/− MEFs, and remained higher than that of wild-type MEFs at other elevated concentrations (Figure 2A). In addition, nuclear translocation of Smad3 occurred at an earlier time point and with higher sensitivity to TGF-β1 in Traf6 −/− MEFs (Figure 2B). A nuclei fractionation assay clearly showed that, upon TGF-β1 treatment, the level of nuclear translocation of Smad2 and Smad3 was elevated in Traf6−/− more than that of the Traf6+/+ MEFs (Figure 2C). To demonstrate the key role of Traf6 in LPS-induced suppression, we conducted TGF-β1/LPS co-treatment experiments using the MEF cells. Traf6−/− MEFs showed much stronger induction of pSmad3 by TGF-β1 treatment than that of Traf6+/+ MEFs, which is consistent with previous results (Figure 2D). Importantly, LPS-induced suppression of Smad3 phosphorylation was observed only in the Traf6+/+ MEFs as co-treated with TGF-β1/LPS, but not in the Traf6−/− MEFs, which implies that Traf6 is playing an inhibitory role for TGF-β signaling in non-transformed MEF cells.

### TRAF6 forms a complex with TβRIII in a TGF-β1-dependent manner

**Figure 3:**
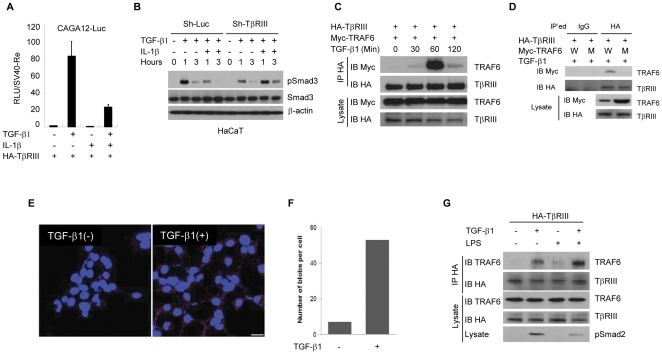
TRAF6 forms a complex with TβRIII in response to TGF-β1. (**A**) CAGA12-Luciferase assays were performed in HepG2 cells. The plasmids encoding HA-TβRIII, TRAF6, CAGA12-Luc, and Renilla-luc reporter gene were transfected as indicated and, on the next day, TGF-β1 (0.4 ng/ml) and/or IL-1β (20 ng/ml) was added for 16 hours. The obtained relative luciferase units(RLU) were normalized by renilla luciferase activities. (**B**) Using the control and TβRIII knock-down HaCaT cells, TGF-β1 (0.4 ng/ml) and/or IL-1β (20 ng/ml) were treated as shown for up to 3 hours. The level of total Smad3 and phospho-Smad3 protein was detected by immunoblotting. For the control of equal loading, β-actin was used. (**C**) HEK293 cells stably expressing HA-TβRIII were transfected with Myc-Traf6 plasmids and then treated with TGF-β1. Cells were harvested at various times and were subjected to immunoprecipitation with anti-HA antibody. Co-immunoprecipitated TRAF6 was detected with anti Myc antibody. (**D**) Complex formation ability between TβRIII and TRAF6 wild-type or the TRAF6 (C85A/H87A) E3 ligase mutant was compared after TGF-β stimulation for an hour. (**E**) According to the manufacturer's protocol, interaction was visualized by *in situ* proximity ligation assay (O-link) with proximity probes directed against TRAF6 and TβRIII using Alexa 555 labeling (red). Endogenous TRAF6 was co-localized with HA-TβRIII along the plasma membrane in the presence of TGF-β (red blobs). Bar = 2.5 µm. (**F**) Quantification of blobs per cell was carried out by semi-automated image analysis using the freeware software BlobFinder V 3.0. (**G**) HA-TβRIII-stably expressing 4T07 mouse mammary cancer cells were treated with TGF-β and LPS for one hour. Co-immunoprecipitation was carried out using anti-HA antibody to query interaction with endogenous TRAF6. The results are representative of three independent experiments.

TβRIII is conventionally described as an accessory receptor that presents the TGF-β ligand to TβRII, thereby boosting TGF-β response [18]. However, recent studies demonstrate that TβRIII has additional roles in regulating receptor internalization through interaction with β-arrestin2, which is involved in terminating TGF-β1 signaling [5]. We confirmed that TβRIII interacts withβ-arrestin2 in a TβRII- and TGF-β-dependent manner (Figure S4A). Interestingly, a recent study showed that β-arrestin1/2 interacts with TRAF6 and suppresses TRAF6-mediated pro-inflammatory responses [19]. Therefore, we explored the possibility that TβRIII is involved in TRAF6-mediated suppression of TGF-β signaling, probably through the common binding partner β-arrestin1/2. We first investigated the relationship between TRAF6 and TβRIII in regulating TGF-β-mediated gene induction by carrying out a CAGA12-Luc reporter gene assay containing promoter with 12 tandem repeats of Smad3 binding sequences. In cells expressing exogenous TβRIII, TGF-β-induced CAGA12-Luc activation was attenuated by IL-1β co-treatment (Figure 3A). TβRIII overexpression markedly enhanced TGF-β1 response, but TRAF6 dose-dependently inhibited TGF-β1 signaling, even in the presence of TβRIII (Figure S5A and S5B). These results suggest that TβRIII plays a significant role in the integration of IL-1β into TGF-β signaling. Interestingly, when the importance of β-arrestin 1/2 was examined, the inhibitory effect of TRAF6 on TGF-β-induced SBE-Luc reporter activity was observed in β-arrestin1/2 −/− MEFs (Figure S4B). This finding suggests that β-arrestin may not play a major role in TRAF6-mediated inhibition of TGF-β signaling. To determine how TβRIII expression affects TRAF6-mediated inhibition of TGF-β signaling, we generated a *TβRIII* knock-down HaCaT cell line by lentiviral knock-down system. RT-PCR results verified that *TβRIII* mRNA expression was almost completely blocked without any changes in *TβRI* and *TβRII* expression (Figure S5C). Interestingly, IL-1βfailed to suppress TGF-β1-induced Smad3 phosphorylation in the *TβRIII* knock-down HaCaT cells (Figure 3B). These results indicate that IL-1β may down-regulate TGF-β signaling through the interactions between TRAF6 and TβRIII proteins. To explore the possibility that the suppression of TGF-β signaling is mediated by enhancing TRAF6/TβRIII interaction, HA-TβRIII and Myc-TRAF6 were expressed in HEK293 cells and treated with TGF-β1 for up to 120 minutes and followed by co-immunoprecipitation assays. Myc-TRAF6 and HA-TβRIII were co-precipitated together only in the TGF-β-treated cells with the most abundant association at 60 minute stimulation (Figure 3C), suggesting that activation of TGF-βsignaling triggers the association between TRAF6 and TβRIII. Next, we tried to determine the importance of TRAF6 E3 ligase activity for the formation of a complex with TβRIII. As compared to wild-type TRAF6, the mutant TRAF6 (C85A/C87H), which has no E3 ligase activity, was unable to interact with TβRIII (Figure 3D). The interaction of TβRIII with TRAF6 was confirmed in the Duolink *in situ* proximity ligation assay (Olink PLA) which enables visualization and quantification of protein interaction when two proteins are at close proximity [20], [21]. HA-TβRIII-stable HEK293 cells were treated or untreated with TGF-β for one hour and followed by immunodetection of endogenous TRAF6 and HA-tagged TβRIII in the OLink PLA. The signals from the proximally localized proteins are amplified as red dots in the OLink PLA. The number of TRAF6/HA-TβRIII complexes per cell was found to be significantly higher in the TGF-β1 treated cells (visualized as red dots in Figure 3E, quantitated in Figure 3F). To test whether TRAF6 and TβRIII association is enhanced by activation of endogenous TRAF6, an HA-TβRIII stably expressing cell line was generated using the 4T07 mouse mammary tumor cell line. The 4T07 cells were used for this experiment because these cells express low to undetectable level of TβRIII which may allow better endogenous TRAF6 and HA-TβRIII interaction without major interference by endogenous TβRIII. TGF-β1 treatment induced interaction between TRAF6 and HA-TβRIII, but co-treatment of cells with LPS and TGF-β1 further enhanced the association of these proteins (Figure 3G). Decreased Smad2 phosphorylation in TGF-β1/LPS co-treated cells suggests that endogenous TRAF6 is involved in LPS mediated inhibition of the TGF-β1/Smad signaling pathway. This result clearly shows that activated TRAF6 migrates to the plasma membrane and avidly forms a complex with TβRIII upon TGF-β treatment.

### Association of TRAF6/TβRIII is regulated by TβRII and TβRI kinase activity

**Figure 4:**
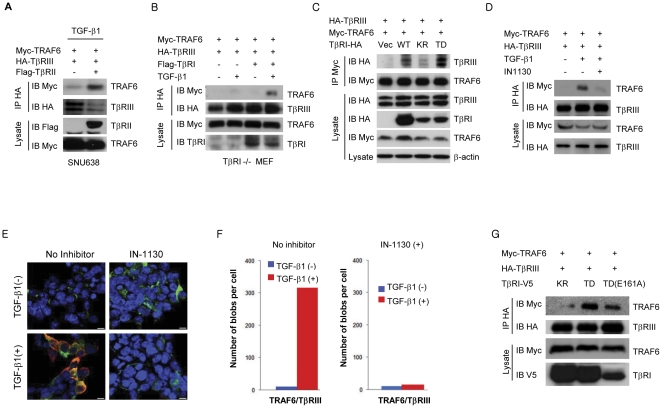
Association of TRAF6/TβRIII is regulated by functional TβRII and TβRI. (**A**) Myc-TRAF6, HA-TβRIII, and/or Flag-TβRII were expressed in SNU638 human gastric cancer cells and treated with TGF-β1 for 60 min. Immunoprecipitation and immunoblotting were carried out to elucidate the requirement of TβRII for TRAF6/TβRIII interaction. (**B**) TRAF6 and TβRIII interaction in TβRI−/− MEFs in the presence or absence of exogenous TβRI expression. (**C**) TRAF6/TβRIII interaction in the presence of wild-type, kinase inactive (KR: K232R), and constitutively active (TD: T204D) TβRI in HEK293 cells. (**D**) Interaction between TRAF6 and TβRIII in the presence of IN-1130 (0.1 µM), a specific inhibitor of TβRI kinase. (**E**) Olink assay for TRAF6 and TβRIII interaction in the presence of TGF-β and/or IN-1130. Transfected cells were counterstained with Alexa 488-labeled HA antibody to HA-TβRIII (green), and the nuclei were stained with Hoechst 33342 (blue). Bar = 2.5 µm. (**F**) The number of *in situ* PLA signals (red blobs) per cell was counted by semi-automated image analysis. (**G**) Co-immunoprecipitation analysis was conducted to elucidate the function of TβRI in TRAF6/TβRIII interaction. TβRI mutants KR, TD, and TD (E161A) mutated in TRAF6 consensus binding motif in TβRI (TD) were used. The results are representative of three independent experiments.

Since the TRAF6/TβRIII complex formed only after TGF-β1 stimulation, we questioned whether TβRII and/or TβRI play a role in TRAF6/TβRIII complex formation. To test the potential TRAF6/TβRIII interaction's dependency on TBRII, SNU638 human gastric cancer cells, which do not express functional TβRII [22], were used in a co-immunoprecipitation assay. TRAF6/TβRIII complex formation was very weak in TGF-β1-treated cells in the absence of functional TβRII (Figure 4A); however, TRAF6 binding to TβRIII was readily detected upon exogenous expression of TβRII and TGF-β1 treatment. Next, we investigated if TβRI is involved in the TRAF6/TβRIII complex formation in *Tgfbr1*−/− MEFs. Whereas trivial interaction of TRAF6/TβRIII was detected in the absence of exogenous TβRI expression, the perceptible association of TRAF6/TβRIII was induced 60 min post treatment of TGF-β1 only in *Tgfbr1*−/− MEFs expressing exogenous TβRI (Figure 4B). This finding implies that TβRI as well as TβRII activation by TGF-β is critical mechanism for the induction of TRAF6/TβRIII complex formation. Therefore, these results indicate that intact TβRII and TβRI expression are important for TRAF6 and TβRIII complex formation upon TGF-β stimulation. To further elucidate the detailed mechanism, we examined whether TβRI kinase activity is required for TRAF6/TβRIII interaction; this was examined by the use of wild-type TβRI, a kinase inactive form of TβRI (K232R; KR), and a constitutively active form of TβRI (T204D; TD), in co-immunoprecipitation assay. As anticipated, the constitutively active form of TβRI (TD) significantly enhanced the interaction between TRAF6 and TβRIII, whereas TβRI wild-type and TβRI (KR) showed respectively moderate and negligible levels of interaction (Figure 4C), suggesting that TβRI kinase activity is important for promoting the TRAF6/TβRIII association. This is further confirmed by demonstrating that the association of TRAF6/TβRIII was largely reduced in cells pre-treated with IN-1130, a TβRI-specific kinase inhibitor [23] (Figure 4D). Additionally, the importance of the TβRI kinase activity was visualized and quantified using the Olink PLA. In the experiment, exogenous TRAF6 and HA-TβRIII were expressed in HEK293 cells and treated with TGF-β1 in the presence or absence of IN-1130. Then, cells were immunostained with anti-Myc (9E10) and anti-HA (Y11) to detect proximally located antigens. As a result, Myc-TRAF6 and HA-TβRIII co-localized in the TGF-β-treated cells, whereas IN-1130-treated cells showed negligible levels of signal, similar to untreated controls (Figs. 4E and 4F). This result validates the importance of TβRI kinase activity in TRAF6 and TβRIII complex formation. Interestingly, there has been an observation that TRAF6 binds to TβRI via the TRAF6 consensus binding motif [24]. We therefore attempted to test whether the consensus binding motif for TRAF6 found on TβRI would affect the interaction between TRAF6 and TβRIII. We performed a co-immunoprecipitation assay with cells expressing TRAF6, TβRIII, and either TβRI (KR), TβRI (TD), or TβRI (TD-E161A), a TβRI with mutation of the TRAF6 consensus binding motif. TRAF6/TβRIII interaction was mostly abrogated by the kinase-inactive TβRI mutant (KR), but only slightly decreased by the TRAF6 consensus binding motif mutant (E161A) of TβRI (TD) (Figure 4G). These results suggest that the TRAF6-TβRIII interaction occurs independently of the TRAF6-TβRI interaction.

### TβRIII is a substrate of TβRI kinase

**Figure 5:**
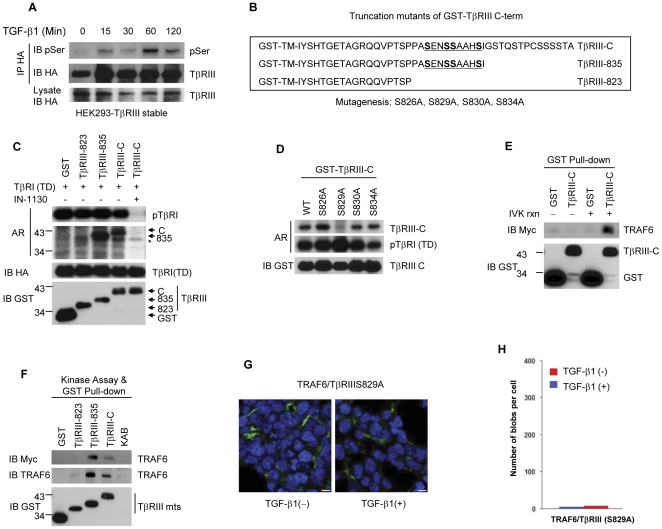
TβRI-mediated phosphorylation of TβRIII at Ser 829 induces preferential binding to TRAF6. (**A**) HEK293 cells stably expressing TβRIII were treated with TGF-β1 over a time course. HA-tagged TβRIII was purified by immunoprecipitation and evaluated the change in serine phosphorylation. (**B**) The transmembrane (TM) and cytoplasmic domains of TβRIII were fused with GST protein. Amino acid sequences for cytoplasmic full-length (TβRIII-C) and deletion mutants (TβRIII-835, TβRIII-823) of GST-TβRIII fusion proteins are illustrated. The potential phosphorylation sites are underlined, and were substituted from Ser to Ala for subsequent experiments. (**C**) Equal amounts of GST-TβRIII-C and deletion mutants were subjected to *in vitro* kinase assay using constitutively active TβRI (TD). IN-1130 (1 µM), a specific inhibitor of TβRI, was added to demonstrate the specificity of kinase reactions. (**D**) The potential phosphorylation sites on GST-TβRIII were mutated to Ala and determined the TβRI (TD)-mediated phosphorylation site of TβRIII. (**E**) A GST pull-down assay was performed using GST-tagged TβRIII-C deletion mutant against cell lysates expressing Myc-TRAF6 proteins. The phosphorylation by TβRI (TD) significantly increased TβRIII and TRAF6 interaction *in vitro*. (**F**) GST-tagged TβRIII-C deletion mutants, subjected to an *in vitro* TβRI (TD) kinase assay, were used to define the region of TβRIII that interacts with TRAF6. (**G**) O-link assay was conducted for visualization of interaction between TRAF6 and TβRIIIS829A in HEK 293T cells. Bar = 2.5 µm. (**H**) The number of *in situ* PLA signals per cell was counted by semi-automated image analysis. The results are representative of three independent experiments.

The cytoplasmic domain of TβRIII has been demonstrated for TβRII interaction and become phosphorylated when TβRII is over-expressed, leading to the dissociation of TβRIII from the TβRII/TβRI complex [25]. Therefore, we speculated that TβRI may be the Ser/Thr kinase that phosphorylates TβRIII upon TGF-β1 stimulation. HEK293 cells stably expressing TβRIII were treated with TGF-β1 for up to 120 minutes, and lysates were immunoprecipitated for HA-TβRIII. Phospho-TβRIII was assessed using a phospho-Serine-specific antibody (Figure 5A). The phospho-form of TβRIII was detected at maximum intensity at 60 minutes after TGF-β1 stimulation, which agreed with the results of TβRIII-TRAF6 interaction shown in Figure 3C. This prompted us to verify whether TβRI directly phosphorylates the cytoplasmic domain of TβRIII. We utilized GST protein-tagged deletion mutants for TβRIII cytoplasmic domain, as illustrated in Figure 5B. HA-TβRI (TD) was expressed in HEK293T cells and then immunoprecipitated with anti-HA antibody for an *in vitro* kinase assay. Consistent with our hypothesis, TβRI (TD) strongly phosphorylated the GST-tagged cytoplasmic domain of TβRIII and the TβRIII-835 mutant, but not the TβRIII-823 mutant (Figure 5C), implying that TβRIII possesses a potential phosphorylation site(s) for TβRI kinase between amino acids 823 and 835. The specific activity of the TβRI (TD) kinase in this assay was determined by addition of 1 µM IN-1130, which almost completely blocked not only auto-phosphorylation of TβRI (TD) but also ablated GST-TβRIII phosphorylation (Figure 5C). Next, to identify the exact phosphorylation site, we prepared GST-TβRIII-C proteins with point mutations at the four serine residues between amino acids 823–835 into alanine, respectively (Figure 5B), and then performed a TβRI (TD) kinase assay. As shown in Figure 5D, TβRI (TD) was able to phosphorylate GST-TβRIII-C and mutants S826A, S830A, and S834A, but was unable to phosphorylate mutant S829A, indicating that residue Ser 829 is a potential phosphorylation target of TβRI (TD). To determine whether phosphorylation of TβRIII may enhance the association of TβRIII with TRAF6, we performed a GST pull-down assay with TβRIII-C-GST fusion proteins that were unphosphorylated or phosphorylated by TβRI (TD) *in vitro*. TRAF6 strongly interacted with TβRIII-C activated by TβRI (TD) (Figure 5E). This result clearly showed that the TβRI-mediated TβRIII phosphorylation is a key mechanism of the TRAF6/TβRIII complex formation. To determine the minimal binding domain of TβRIII, we designed a GST pull-down assay with GST-TβRIII-C deletion mutants. While TRAF6 was able to bind TβRIII-C and the TβRIII-835 mutant phosphorylated by TβRI (TD), it could not bind the TβRIII-823 mutant in which the TβRI (TD)-induced phosphorylation site was deleted (Figure 5F). These results indicate that the TβRIII region between amino acids 823–835, phosphorylated by TβRI (TD), encompasses a site responsible for interaction with TRAF6. We therefore set up an Olink assay to demonstrate the requirement of TβRIII S829 phosphorylation for interaction with TRAF6. In the Olink proximity ligation assay, the interaction between TRAF6 and TβRIII (S829A) was weak and unchanged in response to TGF-β (Figs. 5G and 5H). These results indicate that TβRIII is a novel substrate of TβRI kinase, and that the phosphorylation of TβRIII at S829 by acitivated TβRI kinase is playing a major role for TRAF6/TβRIII interaction.

### TRAF6 dissociates TβRIII from TβRII/TβRI complex

**Figure 6:**
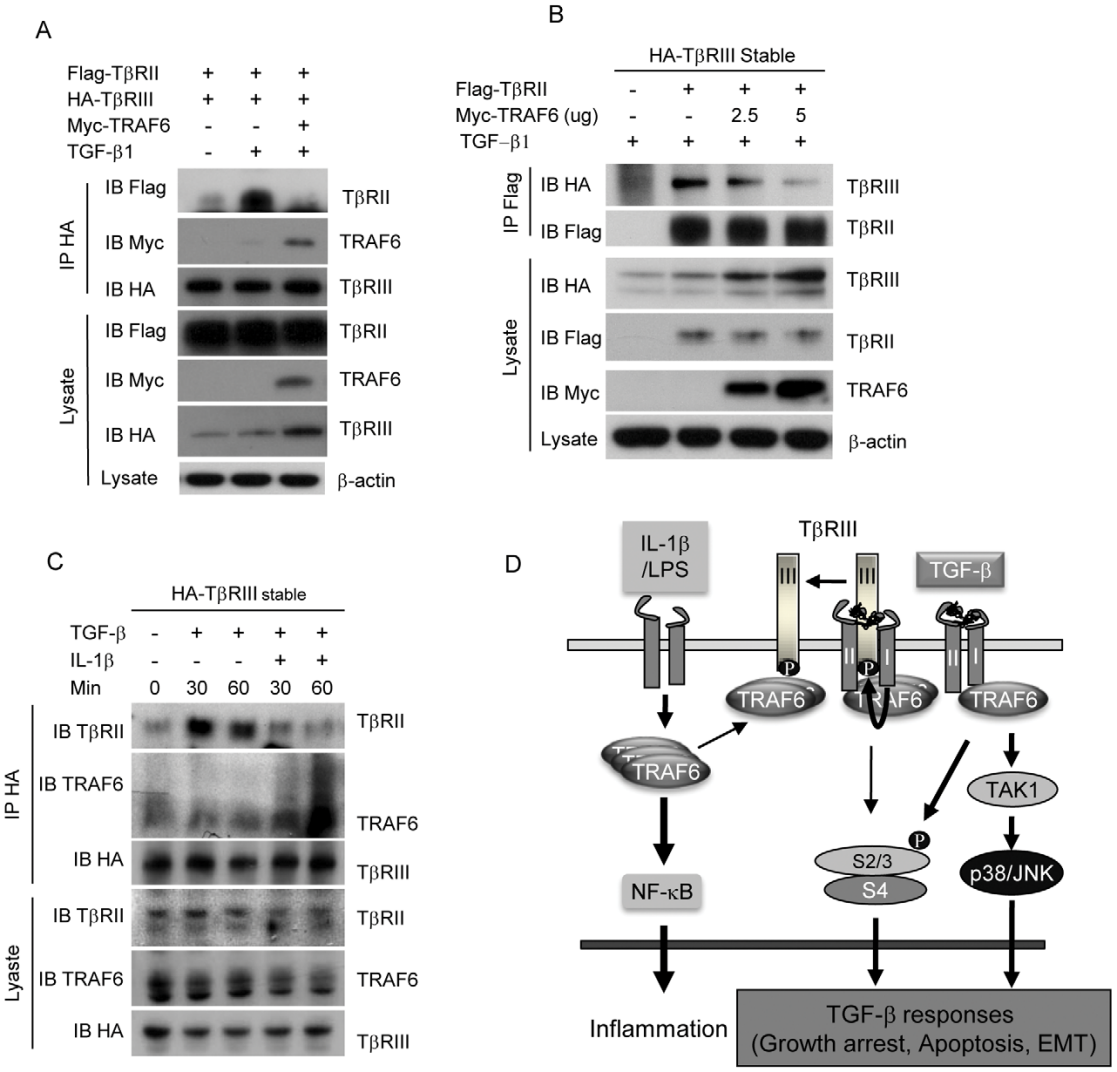
TRAF6 enhances dissociation of TβRIII from TβRII/TβRI complex. (**A**) The interaction between TβRIII and TβRII was enhanced by TGF-β1, and interfered by TRAF6 expression in HEK293 cells. (**B**) Over-expression of TRAF6 in HEK293 HA-TβRIII-stable cells interfere the interaction of Flag-TβRII and HA-TβRIII in a dose-dependent manner. (**C**) HEK293 HA-TβRIII-stable cells were treated as indicated, and were subjected to immunoprecipitation. HA-TβRIII interacted with endogenous TβRII upon TGF-β1 treatment. The level of endogenous TβRII bound to HA-TβRIII was markedly decreased in TGF-β1/IL-1β co-treated cell, while the level of endogenous TRAF6 bound to HA-TβRIII was increased s. These results are representative of three independent experiments. (**D**) A proposed model of IL-1β/LPS-induced suppression of the TGF-β pathway.

In order to elucidate the underlying mechanism by which TRAF6 suppresses TGF-β/Smad signaling through the association with TβRIII, we hypothesized that TRAF6 may disrupt TβRIII/TβRII complex formation, which is critical in boosting cellular TGF-β1 responses [18]. We found that exogenous expression of TRAF6 interrupted TGF-β1-induced TβRIII and TβRII interaction (Figure 6A). Furthermore, the level of TβRII bound to TβRIII was inversely related to the level of TRAF6 expression (Figure 6B). The physiological significance of the influence of TRAF6 on TGF-β signaling was determined by examining endogenous TRAF6 bound to TβRIII in cells treated with TGF-β1 alone or with IL-1β. In the TGF-β1-treated cells, TβRIII/TβRII association persisted up to 60 minutes (Figure 6C). However, in the cells treated with both TGF-β1 and IL-1β, the level of TβRIII-bound TβRII decreased close to the basal level, while the level of TβRIII-associated TRAF6 increased at 60 minutes. We observed the interaction between TβRIII and endogenous TRAF6 in cells treated with only TGF-β (Figure 3G and 6C), which may not be enough to dissociate TβRIII from TβRII complex (Figure 6C). The dissociation is likely to occur only in the case of abundant association of TRAF6 to TβRIII upon LPS or IL-1β co-stimulation (Figure 6C). Taken together, our study suggests a model in which simultaneous treatment with IL-1β/LPS and TGF-β1 provokes robust interaction between TRAF6 and TβR complex while TGF-β alone induces mild interaction of these molecules. Subsequently, IL-1β or LPS suppresses TGF-β/Smad signaling by increasing an interaction between TRAF6 and TβRIII, which is dependent on active TβRI-mediated phosphorylation of TβRIII at Ser829 (Figure 6D). This interaction between TRAF6 and TβRIII is possibly resulted from dissociation of TβRIII from the TβRII/TβRI complex, thereby contributing to the down-regulation of Smad2/3-dependent cellular responses.

Here we have demonstrated that TRAF6, activated by IL-1β or LPS, suppresses TGF-β1/Smad pathways through interaction with TβRIII upon TGF-β1 stimulation. In general, inflammation is tightly regulated and resolved by the induction of anti-inflammatory cytokines [8]. Once this regulatory balance is disturbed, non-specific stimulation and activation of inflammatory cells may lead to increased production and release of potently destructive immunological and inflammatory molecules. TGF-β1 is a pleiotropic cytokine with considerable anti-inflammatory properties. Deficiency of TGF-β1 signaling in the intestine contributes to the development of inflammatory bowel disease, suggesting that maintenance of TGF-β1 signaling is important in regulating immune homeostasis in the intestine [26], [27]. It is well known that inflammatory bowel diseases predispose cancer formation in the intestinal tract. The increased and persistent pro-inflammatory cytokines in the tumor microenvironment play a major role for inducing malignancy [6], [7], [8]. For instance, improper regulation of IL-1β signaling has been shown to potentiate neoplastic risk and ultimately induce tumor progression [8]. In fact, polymorphisms of *IL1B* promoter which enhance IL-1β expression are associated with increased risk of developing human gastric cancer [28], [29]. The study performed in IL-1β knock-out mice suggested that IL-1β enabled local tumor growth and/or metastasis to the lung [30]. In addition, decreased TβRIII expression was closely correlated with tumor progression in various human cancers including breast, lung, prostate, pancreatic, ovarian, and renal cancers [31], supporting the idea that TβRIII-mediated regulation of normal epithelial cells may contribute to prevent tumor progression.

A recent report also showed that Traf6 inhibits Th17 T cell differentiation through the downregulation of Smad signaling [32]. Although the detailed mechanism of this inhibition has not been elucidated in T cells, it supports that Traf6 is partly responsible for inhibiting the TGF-β-induced Smad2/3 signaling pathway *in vivo*. Thus, constitutive activation of TRAF6 by abundant IL-1β in the lesion may contribute to the early stages of tumor development, at least in part, via inhibition of Smad2/3 tumor suppressor pathway.

It is suggested that TRAF6 interacts with TβRI/TβRII complex upon stimulation of TGF-β and mediates activation of p38/JNK MAPKs via TRAF6/TAK1 axies without inhibition of Smad2/3 signaling [24], [33]. We observed that TRAF6 involved in the dissociation of TβRIII from TβRII/I complex in a dose-dependent fashion (Figure 6) and interaction of TRAF6/TβRIII was increased in LPS/TGF-β co-treated cells (Figure 3G). These results suggest a model in which TRAF6 may differently regulate TGF-β-induced Smad2/3 and/or p38/JNK activation depending on the amount of TRAF6 interacting with TGF-βreceptor complex. These regulatory mechanisms are potentially important to determine TGF-β responses such as cell growth arrest, apoptosis, or epithelial-mesenchymal transition.

In summary, our data delineated a key molecular mechanism that IL-β or LPS inhibits anti-inflammatory TGF-β/Smad signaling through TRAF6 and TβRIII association in TGF-β- and TβRI kinase-regulated manner. Although the antagonistic effects of IL-1β/LPS on TGF-β1 signaling may occur at multiple molecular levels, a fine modulation of the TGF-β receptors may critically affect cell fates by changing the kinetics of Smad2/3 phosphorylation. By this mechanism, IL-1β may potentiate inflammatory responses, and chronic inflammation may further contribute to tumor progression by suppressing the TGF-β anti-inflammatory and tumor suppressor pathways.

## Materials and Methods

### Cell culture and reagents

HepG2, HEK293, HEK293T, 67NR, 4TO7, and 4T1 cells were obtained from ATCC and HaCaT cells were kindly provided by Dr. Stuart Yuspa (National Cancer Institute, Bethesda, MD). TRAF6-deficient mouse embryonic fibroblasts and b-arrestin-deficient MEFs were gifted from J. Inoue (Keio University) and R. Lefkowitz (Duke University), respectively. FaO cell were also kindly gifted by Dr. I. Lim (Aju university). HaCaT, HepG2, FaO, HEK293, and HEK293T cells were grown in DMEM, and 67NR and 4T07 breast cancer cell lines were grown in RPMI supplemented with 10% (vol/vol) FBS, penicillin (100 units/ml), and streptomycin (100 µg/ml; Invitrogen, Rockville, MD). Cells with stable expression of TβRIII were generated by transfection with pCDNA3.1-HA-TβRIII and then selected as a pool in selection medium containing neomycin. Antibodies specific for hemagglutinin (Y-11 or F-7), Myc (9E10), TRAF6 (H-274), TβRII (C-16), and TβRI (V-22) were purchased from Santa Cruz; antibodies to Flag (anti-Flag; M2) and anti-β-actin (AC-15) were from Sigma; specific antibodies for p-Smad2, p-Smad3, and total Smad2 were from Cell Signaling; anti-Smad3 and anti-phosphoserine specific antibodies were from Zymed Laboratories.

### TUNEL assay

TGF-β-induced cell death in FaO cells was demonstrated using In Situ Cell Death Detection Kit Fluorescein (Roche) according to manufacturer's protocols, which can detect single- and double-stranded DNA breaks that arise at early stages of apoptosis.

### DNA Fragmentation assay

Following treatment of FaO cells with TGF-β and/or LPS for overnight, all of the adherent and floating cells were collected and subjected to genomic DNA preparation. The collected cells were resuspended in 400 µl extraction buffer (0.5% SDS, 0.1 M NaCl, 0.01 M EDTA, 0.02 M Tris-HCl (pH 7.6), and proteinase K (100 µg/ml)) and followed by incubation at 60°C for 2 hours. The lysates were resuspended with 200 µl of 6 M NaCl, mixed thoroughly, and placed on ice for 5 minutes. After spin down the lysates for 10 minutes at 13,000 rpm, the 400 µl supernatants were transferred and mixed with 100% ethanol to precipitate genomic DNA. The precipitated DNA was harvested and dissolved in TE buffer. Finally, the DNA was incubated for 1–2 hours at 56°C, and measured DNA concentration and loaded in 1% agarose gel with equal amount of genomic DNA.

### Immunofluorescence microscopy

Wild-type and Traf6-deficient MEF cells were grown on 22-mm glass coverslips in a CO_2_-humidified incubator for 24 h. Smad3 was detected by incubation at 4°C with anti-Smad3 rabbit polyclonal (Zymed Laboratories Inc.). The images were acquired with Nikon microscope by using MetaMorph software.

### Nuclear fractionation and quantification

Nuclear and cytoplasmic fractionations were performed according to previously described methods [34]. Density of detected Smad2 and Smad3 was quantitated using ImageJ software and marked as a fold change.

### Knock-down and over-expression using Lentiviral infection system

Knockdown of endogenous Traf6 and TβRIII gene expression was carried out using the lentivirus shRNA expression system and experimental method as previously described [10]. The target sequence of mouse *traf6* nucleotide 618–637 (AGCTGTCCTCTGGCAAATATC), human *TGFBR3* nucleotide 1878–1897 (ATGGTGTGGTCTACTATAA), or control Luciferase nucleotide was used. For over-expression, the TRAF6 gene was introduced into pCAG-MCS-W Lentiviral vector and transfected for viral packaging.

### In vitro site-directed mutagenesis

In order to generate mutant constructs (S826A, S829A, S830A, and S834A) for C-term of TβRIII (TβRIII-C), we performed in vitro site-directed mutagenesis reaction by employing QuickChange Site-directed mutagenesis kit (Stratagene). We designed specific primers for each mutant construct (supplementary data) according to the manufacturer's protocol.

### *In Vitro* Kinase Assay

The TβRI kinase assay was performed as described [23] and GST-TβRIII deletion mutant proteins were prepared according to the manufacturer's protocol (Amersham Bioscience). The truncation mutants of TβRIII-835 and TβRIII-823 were generated by introducing termination codon, according to the instruction of site-directed mutagenesis kit (Stratagene). The phospho-labeled proteins were detected by autoradiography.

### Proximity ligation assay (PLA)

PLA was performed to visualize protein–protein interactions using the reagent kit from Olink Bioscience. In brief, oligonucleotide conjugated ‘probe’ antibodies are directed against primary antibodies. Annealing of the ‘probes’ occurs when the target proteins are in close proximity, which initiates the amplification of a Texas red reporter signal [21]. After TβRIII-myc and TRAF6-myc transfection, HEK 293T cells were treated with TGF-β1 for 1 hr , 0.1 µM IN-1130 for 2 hr. Cells were washed with chilled PBS and fixed with acetone for 20 mins, and incubated overnight with mouse anti-myc (1∶100, Santa Cruz, sc-40) and rabbit anti-HA (1∶100, Santa Cruz, sc-805) at 4°C. Proximity ligation was performed according to the manufacturer's protocol using the Duolink Detection Kit with PLA PLUS and MINUS Probes for mouse and rabbit (Olink Bioscience). Hoechst stain was included in the Duolink Detection Kit while anti-rabbit Alexa 488 (1∶500; Invitrogen Inc.) was added during the detection reaction. Specimens were mounted with Vecta-shield mounting media (Vector Laboratories) and examined with and confocal microscope (Carl Zeiss, LSM 710). The number of *in situ* PLA signals per cell was counted by semi-automated image analysis using the freeware software BlobFinder V 3.0.

## Supporting Information

Figure S1 **TRAF6 mediates IL-1β/LPS signaling for suppression of TGF-β/Smad signaling.** (**A**) HaCaT cells were treated with TGF-β, IL-1β, and/or LPS as indicated. Altered pSmad2/3 levels demonstrated the mitigated TGF-β responses upon IL-1β or LPS co-treatment. (**B**) IL-1β-induced NF-κB promoter activation is blocked by TAK1 (K63W) expression. (**C**) Smad7 messenger RNA expression is not affected by exogenous expression of TRAF6 or TAK1 (K63W) or addition of IL-1β for 1 hour in HepG2 cells. (**D**) TRAF6-mediated suppression of SBE-promoter activity following TGF-β addition was examined in HepG2 cells which are knocked down smad7 expression by lenti-shSmad7 or pLKO control. (**E**) 67NR cells were infected with lentivirus containing either control sh-Luciferase (sh-Luc) or sh-TRAF6 and then selected by Puromycin (2 µg/ml) for a week. TRAF6 protein expression was ablated in sh-TRAF6 infected cells, while β-actin expression was unaffected. Cells were examined to compare the level of phospho-Smad2/3 in response to TGF-β1 (0.4 ng/ml) with or without LPS (50 ng/ml). (**F**) Myc-TRAF6 or GFP over-expressing HaCaT cells were treated with TGF-β for up to 6 hours and then harvested for RT-PCR. TGF-β target genes (*CDKN1A* and *SMAD6*) were compared in control and exogenous TRAF6-expressing cells.
(TIF)

Figure S2 **LPS antagonized TGF-β-induced apoptosis of FaO cells in a NF-κB independent manner.** (A) FaO cells were treated with TGF-β or TGF-β/LPS for the time as indicated (TGF-β1(5 ng/ml), LPS (50 ng/ml)). The change of pSmad3 level was shown upon TGF-β alone or simultaneous addition of TGF-β/LPS. (B) FaO cells were infected with either adenoviral LacZ or IκBαsr and treated with TGF-β alone or together with LPS up to 8 hours. Cells were harvested to compare the induction of cleaved caspase-3. (C) FaO cells were treated with TGF-β and/or LPS as indicated for overnight followed by TUNEL assay. The morphologic difference was shown in photographs with phase contrast (PC) and fluorescent microscopy (TUNEL) at 40×. (D) FaO cells were stimulated with TGF-β and/or LPS. The adherent and floating cells were collected and subjected to DNA fragmentation assay. Equal amount of genomic DNA was loaded.
(TIF)

Figure S3 **The inhibitory role of Traf6 in wild-type and Traf6 −/− MEFs for TGF-β/Smad signaling** Wild-type and Traf6 −/− MEFs were grown in 6-well plates and harvested for total RNA or lysed for immunoblotting. (**A, left**) RT-PCR was performed to show expression of endogenous *Tgfbr1*, *2*, *3*, and *Traf6* messenger RNA. (**A, right**) Immunoblotting was carried out for TβRI, TβRII, and Traf6 proteins. β-actin was used as a loading control. (**B**) SBE-Luc reporter gene assay was performed in wild-type and *Traf6* −/− MEFs with or without TGF-β1 treatment (0.4 ng/ml). The SBE promoter-derived luciferase activity was normalized by co-transfected β-galactosidase and plotted as a fold induction by setting 1wtih unstimulated control cells.
(TIF)

Figure S4 **β-arrestin2 interacts with TβRIII in a TGF-β and TβRII dependent manner, but does not play a key role for Traf6-mediated inhibition of TGF-β signaling.** (**A**) A schematic diagram for Myc-tagged β-arrestin2 deletion mutants that were used in subsequent co-immunoprecipitation assays (upper). The full-length and N-terminal portion of β-arrestin2 show a capacity for TβRIII binding in a manner that is TβRII- (left) and TGF-β1- (right) dependent. (**B**) SBE-Luciferase assay shows that TRAF6 inhibits TGF-β signaling in β-arrestin2 +/+, β-arrestin2 −/−, and β-arrestin1/2 −/− MEFs.
(TIF)

Figure S5 **Suppression of TβRIII-mediated SBE promoter activation by TRAF6 and knock-down of TβRIII in HaCaT cells** (A) SBE-Luciferase assays were performed in HepG2 cells. The plasmids encoding HA-TβRIII, TRAF6, SBE-Luc, and β-galactosidase gene were transfected as indicated and, on the next day, followed by TGF-β1 (0.4 ng/ml) addition for 16 hours. The obtained relative luciferase units (RLU) were normalized by β-galactosidase activities. (B) TRAF6 mediated suppression of TGF-β signaling was further examined in the presence of TβRIII with increasing amount of TRAF6 expression. (**C**) HaCaT cells were infected with lentivirus encoding sh-TβRIII or control sh-*Luciferase*. RT-PCR was carried out to demonstrate the knock-down of TβRIII mRNA. *TGFBR1* and *TGFBR2* mRNA expression was unaffected by sh-TβRIII expression. GAPDH indicated that equal amount of RNA was used in RT-PCR.
(TIF)
