# Supplementary figures and images for: TRAF6 Mediates IL-1β/LPS-Induced Suppression of TGF-β Signaling through Its Interaction with the Type III TGF-β Receptor
Source: PLoS One. 2012 Mar 12;7(3):e32705. doi: 10.1371/journal.pone.0032705 (PMC3299683; doi:10.1371/journal.pone.0032705)

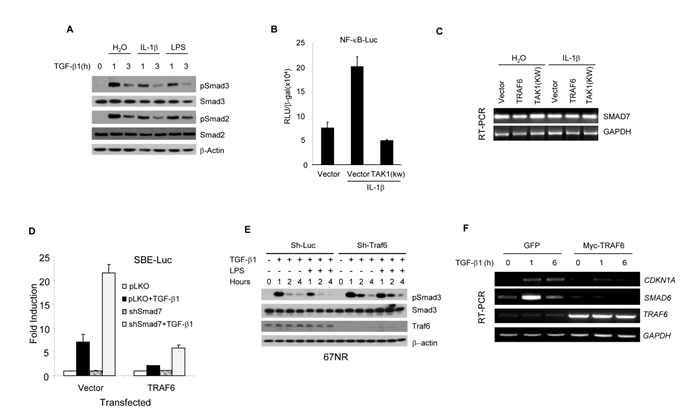

Supplement: Figure S1 — TRAF6 mediates IL-1β/LPS signaling for suppression of TGF-β/Smad signaling. (A) HaCaT cells were treated with TGF-β, IL-1β, and/or LPS as indicated. Altered pSmad2/3 levels demonstrated the mitigated TGF-β responses upon IL-1β or LPS co-treatment. (B) IL-1β-induced NF-κB promoter activation is blocked by TAK1 (K63W) expression. (C) Smad7 messenger RNA expression is not affected by exogenous expression of TRAF6 or TAK1 (K63W) or addition of IL-1β for 1 hour in HepG2 cells. (D) TRAF6-mediated suppression of SBE-promoter activity following TGF-β addition was examined in HepG2 cells which are knocked down smad7 expression by lenti-shSmad7 or pLKO control. (E) 67NR cells were infected with lentivirus containing either control sh-Luciferase (sh-Luc) or sh-TRAF6 and then selected by Puromycin (2 µg/ml) for a week. TRAF6 protein expression was ablated in sh-TRAF6 infected cells, while β-actin expression was unaffected. Cells were examined to compare the level of phospho-Smad2/3 in response to TGF-β1 (0.4 ng/ml) with or without LPS (50 ng/ml). (F) Myc-TRAF6 or GFP over-expressing HaCaT cells were treated with TGF-β for up to 6 hours and then harvested for RT-PCR. TGF-β target genes (CDKN1A and SMAD6) were compared in control and exogenous TRAF6-expressing cells. (TIF) [file pone.0032705.s001.tif]

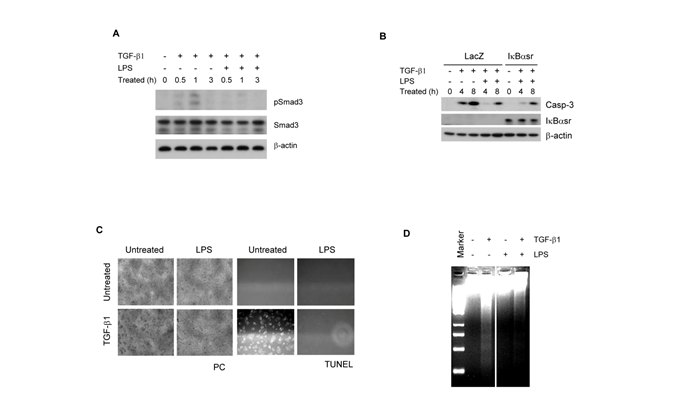

Supplement: Figure S2 — LPS antagonized TGF-β-induced apoptosis of FaO cells in a NF-κB independent manner. (A) FaO cells were treated with TGF-β or TGF-β/LPS for the time as indicated (TGF-β1(5 ng/ml), LPS (50 ng/ml)). The change of pSmad3 level was shown upon TGF-β alone or simultaneous addition of TGF-β/LPS. (B) FaO cells were infected with either adenoviral LacZ or IκBαsr and treated with TGF-β alone or together with LPS up to 8 hours. Cells were harvested to compare the induction of cleaved caspase-3. (C) FaO cells were treated with TGF-β and/or LPS as indicated for overnight followed by TUNEL assay. The morphologic difference was shown in photographs with phase contrast (PC) and fluorescent microscopy (TUNEL) at 40×. (D) FaO cells were stimulated with TGF-β and/or LPS. The adherent and floating cells were collected and subjected to DNA fragmentation assay. Equal amount of genomic DNA was loaded. (TIF) [file pone.0032705.s002.tif]

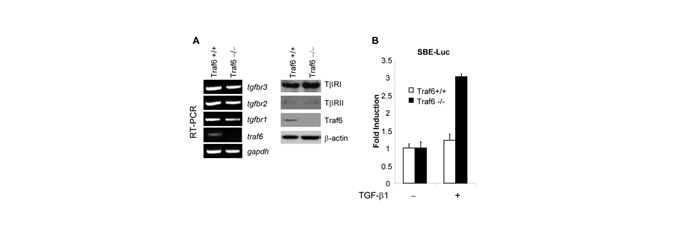

Supplement: Figure S3 — The inhibitory role of Traf6 in wild-type and Traf6 −/− MEFs for TGF-β/Smad signaling Wild-type and Traf6 −/− MEFs were grown in 6-well plates and harvested for total RNA or lysed for immunoblotting. (A, left) RT-PCR was performed to show expression of endogenous Tgfbr1, 2, 3, and Traf6 messenger RNA. (A, right) Immunoblotting was carried out for TβRI, TβRII, and Traf6 proteins. β-actin was used as a loading control. (B) SBE-Luc reporter gene assay was performed in wild-type and Traf6 −/− MEFs with or without TGF-β1 treatment (0.4 ng/ml). The SBE promoter-derived luciferase activity was normalized by co-transfected β-galactosidase and plotted as a fold induction by setting 1wtih unstimulated control cells. (TIF) [file pone.0032705.s003.tif]

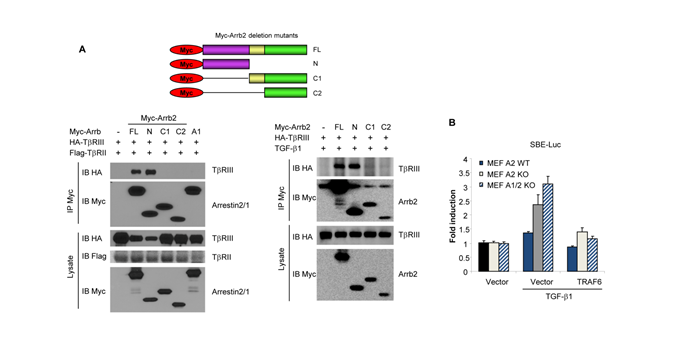

Supplement: Figure S4 — β-arrestin2 interacts with TβRIII in a TGF-β and TβRII dependent manner, but does not play a key role for Traf6-mediated inhibition of TGF-β signaling. (A) A schematic diagram for Myc-tagged β-arrestin2 deletion mutants that were used in subsequent co-immunoprecipitation assays (upper). The full-length and N-terminal portion of β-arrestin2 show a capacity for TβRIII binding in a manner that is TβRII- (left) and TGF-β1- (right) dependent. (B) SBE-Luciferase assay shows that TRAF6 inhibits TGF-β signaling in β-arrestin2 +/+, β-arrestin2 −/−, and β-arrestin1/2 −/− MEFs. (TIF) [file pone.0032705.s004.tif]

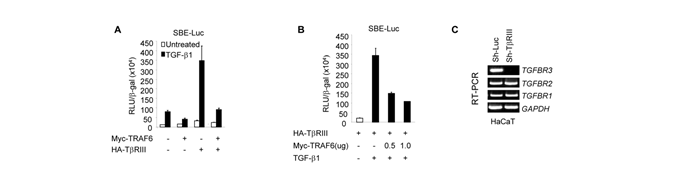

Supplement: Figure S5 — Suppression of TβRIII-mediated SBE promoter activation by TRAF6 and knock-down of TβRIII in HaCaT cells (A) SBE-Luciferase assays were performed in HepG2 cells. The plasmids encoding HA-TβRIII, TRAF6, SBE-Luc, and β-galactosidase gene were transfected as indicated and, on the next day, followed by TGF-β1 (0.4 ng/ml) addition for 16 hours. The obtained relative luciferase units (RLU) were normalized by β-galactosidase activities. (B) TRAF6 mediated suppression of TGF-β signaling was further examined in the presence of TβRIII with increasing amount of TRAF6 expression. (C) HaCaT cells were infected with lentivirus encoding sh-TβRIII or control sh-Luciferase. RT-PCR was carried out to demonstrate the knock-down of TβRIII mRNA. TGFBR1 and TGFBR2 mRNA expression was unaffected by sh-TβRIII expression. GAPDH indicated that equal amount of RNA was used in RT-PCR. (TIF) [file pone.0032705.s005.tif]
